# Supplementary material for: Australian hospital paediatricians and nurses’ perspectives and practices for influenza vaccine delivery in children with medical comorbidities
Source: PLoS One. 2022 Dec 12;17(12):e0277874. doi: 10.1371/journal.pone.0277874 (PMC9744269; doi:10.1371/journal.pone.0277874)
Supplement: S1 Appendix — (DOCX) [file pone.0277874.s001.docx]

# Appendix 1: Clinical Provider Interview and Discussion Group Session Guide

**Introduction for interviewee**

This discussion group session is aimed at better understanding the influenza vaccination program for your paediatric patients with medical conditions. Specifically, your understanding, experiences, and attitudes towards the influenza vaccine, in particular in the context of your department/s and/or hospital.

This is a qualitative study, with open-ended questions. This document serves as a guide for the session and not a verbatim script of what the interviewer will be saying. Not all questions will be asked during all sessions and not necessarily in the same sequence. Potential areas that we may want to use for follow-up questions (i.e. probes) are listed under the main questions.

**Introductory script**

- Thank the participants for agreeing to take part in this study.
- Introduce the study and briefly discuss its purpose.
- Discuss the role of the interviewer: to raise topics for discussion and then to listen as the participants shares their views and experiences.
- Reassure the participants that they are free to talk about any aspect of their experience or attitudes. There are no right or wrong or even typical answers to any of the questions that we will discuss.
- Remind the participants that, with their permission, the session will be audio-taped.
- Reassure confidentiality and the participants’ right to stop the session at any time.
- Clarify that the discussion session will take approximately 30 minutes to 1 hour.
- Ask whether the participants have any questions before we begin.
- Patient models will be used to discuss

# Discussion Group Sessions Questions

1. In broad terms, can anyone describe the current influenza vaccination program at your hospital for your outpatients (if any)?
   1. If the program run by your department/clinic or by the hospital as a whole?
   2. Do you have or are aware of a program for inpatients?
   3. Do you have a direct role in either of these programs?
   4. Process**:** Where do patients go to receive the vaccine, how do you request a vaccine
      1. Do you see any issues with the process: Time, paperwork, availability?
      2. What about recording a vaccination being given?
2. How do you view the success of this program each year?
   1. Are there any specific barriers for the programs each year?
   2. How do parents usually react to the program each year?

**Introduction of a theoretical model patient**

1. Does this model patient reflect the kind of patient you would typically see in the your department/clinic?
   1. If not, why not?
2. What risk do you believe influenza would be to this patient to be?
   1. Risk of infection, hospitalisation, ICU and mortality
3. What is the likelihood this patient would receive the influenza vaccination each season?
   1. What is the likelihood they would receive the vaccine at your department/clinic?
   2. How likely are you to recommend the vaccine to this patient?
4. Now with this patient can you describe the journey they would go on to receive the influenza vaccine at your department/clinic
   1. Are they anything specific with this patient that would prevent them from being vaccinated?
   2. Are there any physical/temporal barriers to prevent them from receiving the vaccine?
   3. Are there any procedural barriers that would prevent them from receiving the vaccine at your department/clinic?
