# Supplementary material for: Australian hospital paediatricians and nurses’ perspectives and practices for influenza vaccine delivery in children with medical comorbidities
Source: PLoS One. 2022 Dec 12;17(12):e0277874. doi: 10.1371/journal.pone.0277874 (PMC9744269; doi:10.1371/journal.pone.0277874)
Supplement: Attachment — Submitted filename: Reviewer response 20_09.docx [file pone.0277874.s002.docx]

20th of September 2022

Dear reviewers,

Thank you for your time in reviewing our submission. Please find below the details of our edits to the manuscript below and the new version of the manuscript uploaded.

We have clarified the funding of influenza vaccination in Australia and how this pertains to cocooning vaccination on lines 483 to 487 based on reviewer 1’s comments. Additionally, we have included further limitations as detailed by reviewer 2’s comments in lines 507 to 516. We have included further details about the interviews and discussion groups in the methods section in lines 139 to 153. We have modified the funding statement as per the publisher’s request on lines 36 to 47.

We have updated our reference list based on our edits from reviewers’ comments. These two new references include

19.	The Australian Technical Advisory Group on Immunisation. ATAGI advice on seasonal influenza vaccines in 2022: The Commonwealth of Australia; 2022 [cited 2022 10/08/2022]. Available from: https://www.health.gov.au/sites/default/files/documents/2022/02/atagi-advice-on-seasonal-influenza-vaccines-in-2022.pdf.

20.	Queensland Government. Influenza (flu) vaccinations 2022: The State of Queensland; 2022. Available from: https://www.qld.gov.au/health/conditions/immunisation/free-influenza-vaccinations-2022.

Daniel Norman, BSc, MInfDis, MPH PhD

Senior Research Officer, National Centre for Immunisation Research and Surveillance
Corresponding author
Postal address: NCIRS, Kids Research, Sydney Children’s Hospitals Network, Cnr Hawkesbury Rd & Hainsworth St, Westmead Locked Bag 4001, Australia
Telephone: (+61) 4229 9161
Email: da.norman@outlook.com
